# Thermal Imaging of Exercise-Associated Skin Temperature Changes in Swimmers Subjected to 2-min Intensive Exercise on a VASA Swim Bench Ergometer

**DOI:** 10.3390/ijerph18126493

**Published:** 2021-06-16

**Authors:** Anna Knyszyńska, Aleksandra Radecka, Anna Lubkowska

**Affiliations:** Department of Functional Diagnostics and Physical Medicine, Pomeranian Medical University, 71-210 Szczecin, Poland; aleksandra.radecka@pum.edu.pl (A.R.); anna.lubkowska@pum.edu.pl (A.L.)

**Keywords:** thermal imaging, skin temperature, swimming training

## Abstract

An important element of swimming training is the improvement of muscle strength and the technique of swimming movements on dry land. The heat generated by the muscles involved in the effort contributes to a change in the temperature of the skin surface, which can be assessed by the IRT method. The aim of the study was to assess the symmetry and dynamics of changes in surface temperatures of selected areas of the body in swimmers after exercise on a swimming ergometer with the use of IRT. A total of 12 swimmers (aged 19 ± 1.3 years) completed a two-minute stress test (front crawl swimming movements) using a VASA Swim Ergometer, with a load of 5. Using an IRT camera (FLIR E60), postexercise changes in back and upper limbs surface temperature in relation to the resting values were observed. After exercise, the temperature value of all assessed areas decreased, apparently in the area of the back and the back of the arms, returning to the baseline values after 12 min of observation. There was no asymmetry in mean temperature values between the right and left upper limbs. IRT is a noninvasive and sensitive tool for the individual analysis of changes in body surface temperature in swimmers after training on dry land.

## 1. Introduction

Swimming performance is considered to be largely dependent on muscular power and strength, the latter being identified as the main determinant of success in competitive swimming. The strength of the muscles of the upper body parts is particularly important, as they generate most of the driving forces and are responsible for the speed of swimming [1]. Strength training is therefore an important element of swimming training [2,3], but it should be remembered that the movements used during training and exercise testing should be biomechanically similar to the movements performed during the competition [4]. The so-called dry-land swimming training consists of a series of exercises focused on dynamic force and a specific movement performed with constant or variable force. The maximum strength value that can be achieved is determined individually for each athlete. Initially, particular emphasis is placed on shaping the level of control and technique, paying special attention to symmetrization of the arm movement. One of the devices that enables the mapping and improvement of swimming movements during dry-land training is the VASA ERGOMETER [5]. It is well known that symmetrical application of force and equal loading of the right and left sides of the body have a positive effect on swimming performance, especially by reducing resistance and reducing speed variations in the cycle [6]. In fact, about 96% of the human population shows a noticeable level of central asymmetry in central control of movement, including force generation and precision [7], which can negatively affect exercise performance, mainly in cyclical and continuous sports [8,9].

The optimization of training in modern competitive sports is based on the constant monitoring of training effects and the response of the athlete’s body to training stimuli. This allows for the maximum use of the athlete’s physical capacity potential and individual selection of training tasks and loads, resulting in better sports results [10]. An important aspect of such an assessment is the analysis of muscle involvement in the effort, and in some sports, including swimming, the symmetry of their work is particularly essential. Muscle activity during their work is most often assessed using electromyography (EMG) methods, but their use requires specialized equipment, as well as the ability to process and interpret electrical potential records. Moreover, in the case of needle electromyography, we are dealing with a more precise method, but it is also invasive for the examined person. Undoubtedly, EMG is the most accurate tool for assessing muscle activation, especially in clinical trials. However, assessing sports performance requires a quick method that can be carried out in a screening mode. Therefore, other ways of assessing muscle involvement in exercise are being sought [11,12]. The measurement of the energy and metabolic activity of certain muscle groups is a significant technical problem. Modern methods of assessing the energy activity of muscles during human movement are based on the indirect calorimetry method, which only provides information about the total energy demand of the body [13]. A large amount of chemical energy in the muscles is converted into heat during muscle contractions. The total heat of muscles is the sum of their static heat, shortening, and regeneration [14]. Higher metabolic activity and blood supply to the muscle tissue during physical activity cause a significant increase in muscle temperature, and the generated thermal energy is transferred to adjacent tissues, including the skin, for its elimination via thermoregulation mechanisms [15]. Therefore, one of the noninvasive and quantitative methods of assessing training effects that is increasingly used in sports medicine, which takes into account the response of the athlete’s body to the physical work assigned to him/her, and the possibility of objectively assessing it in terms of correct physiological reactions, especially thermoregulatory ones, is infrared thermography (IRT) [15,16,17,18,19,20,21,22,23,24,25]. IRT is a safe, noninvasive, and low-cost technique that allows for the rapid and noncontact recording of the irradiated energy that is released from the body. All these factors make it possible to obtain test results multiple times and repetitively, without exposing the patient to unpleasant stimuli [26]. Moreover, correlations between the changes in body surface temperature recorded by IRT and the parameters of the EMG recording of muscle activity during exercise were confirmed [27]. In addition, the assessment of the symmetry of biomechanics of movements during various forms of physical activity, possible thanks to thermal imaging, is important for sportspersons, coaches, and medical personnel due to the possible relationship with work efficiency, injury risk assessment, or post-traumatic changes [26,28,29]. To our knowledge, only a few reports present results relating to the possibility of assessing muscular effort and the efficiency of thermoregulatory mechanisms based on the use of IRT. The nature of thermal changes in the body surface over the working muscles during and after exercise was assessed in handball players, rowers [30], basketball players [31], runners [32], cyclists [33], kickboxing, and Muay Thai fighters [34]. Single studies have looked at the use of IRT in assessing symmetry of muscle involvement in swimming [13]. It has been shown that the forces generated by swimmers during dry-land swimming training are closely related to the ability to use the same force values in the water environment [35,36]. While swimming in real conditions, the water environment significantly affects the temperature of the body surface and its postexercise changes, making it impossible to objectively assess thermal symmetry as a result of muscular effort. Therefore, the aim of the study was the thermovision assessment of the symmetry and dynamics of changes in surface temperature of selected areas of the swimmers’ body as a response to muscle involvement during a two-minute intensive exercise on a swimming ergometer.

## 2. Materials and Methods

### 2.1. Study Group

The study group consisted of 12 men practicing regular freestyle swimming at the Miejski Klub Pływacki (Municipal Swimming Club) in Szczecin, Poland. The mean age of the subjects was 19 ± 1.3 years, and the mean Body Mass Index (BMI) was 21.9 ± 1.19 kg/m^2^. The average swimming training experience was 11–16 years. The number of training sessions varied from 4 to 6 per week.

Each athlete provided written consent before participating in the research, according to the Declaration of Helsinki. The study was approved by the Local Ethics Committee of the Pomeranian Medical University (Ref. No. KB-0012/36/13). All participants were healthy and not injured.

The participants were instructed on how to prepare for the study in accordance with the guidelines Thermographic Imaging in Sports and Exercise Medicine (TISEM) [10].

### 2.2. Procedures

The research procedures were carried out in a closed training room belonging to the Floating Arena 50 m swimming pool of the Municipal Sports, Recreation, and Rehabilitation Centre in Szczecin, Poland. The research was carried out in the hours corresponding to the afternoon training hours, i.e., between 15:00 and 17:00, with two swimmers being tested per day, at the same times of the day. The survey data necessary to characterize the group were collected from all the surveyed swimmers. Subsequently, body height and weight were measured in all subjects with the use of a mechanical column scale (Seca 711/220) with a stadiometer, and the BMI was calculated to characterize the subjects.

At a later stage of the research, the swimmers individually took the exercise test using the VASA Swim Bench Ergometer (Vasa Inc., Essex Junction, VT, USA). It is a device used during “dry-land” swimming training, which the tested swimmers systematically use in training cycles. Its design is based on a flywheel mechanism that allows to generate a load on a 7-point scale of resistance. You can adjust airflow resistance by changing the opening of the damper door on the front of the Vasa Ergometer. The lowest setting, “1” (door fully closed), provides the least resistance, and setting “7” (door fully open) provides the most resistance. Setting 1 is similar to going with the current, and setting 7 is similar to going against a strong current. In the test used, the load was selected at level 5, which is the load most used during the training of the evaluated swimmers. The movements performed during such training imitate symmetrical swimming movements of double arms in the freestyle position and involve the muscle groups involved in traditional water swimming [5]: shoulder girdle muscles, upper back muscles, upper limb muscles. The lower back muscles and the abdominal muscles, which ensure stabilization, were involved in the effort to a lesser extent. The lower limbs were not involved in the effort, and their proximal parts were leaning on the bench. The respondents did not need time to familiarize themselves with the ergometer because it is a device on which they exercise systematically as part of a warm-up or training. The duration of the exercise test, during which the swimmers had to maintain maximum arm speed using freestyle technique, was 2 min for each swimmer. Such assumptions correspond to the efforts made during the 200 m freestyle competition in which the subjects participate most often. The ergometer was calibrated before each subsequent trial. In order to assess the thermal response to physical exercise, before the exercise test and at the next designated measurement points after its completion, 5 thermal images in the anatomical position in the anteroposterior (AP) projections were taken for each of the swimmers.

For each of the subjects, a series of thermographic measurements was performed according to the following scheme:

- T_befor_—before the exercise test on a swimming ergometer;
- T_after_—immediately after the exercise test lasting 2 min;
- T_3min_—3 min after the end of the exercise test;
- T_6min_—6 min after the end of the exercise test;
- T_9min_—9 min after the end of the exercise test;
- T_12min_—12 min after the end of the exercise test.

**Figure 1:**
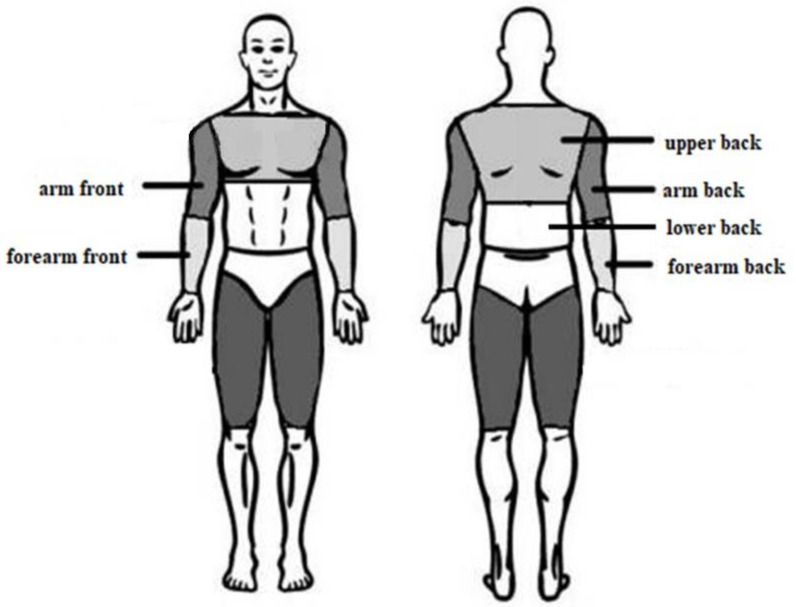
Analyzed body surfaces.

For thermographic assessment, the exposed areas of the body surface were selected, corresponding to the topographic position of the muscles most intensively involved during freestyle swimming training and during physical exercise on a swimming ergometer using the freestyle technique, i.e., the arm area (A), forearm area (FR), upper back area (UB), and lower back area (LB). The chest and abdomen areas were not included in the analysis due to the position of the respondents during the stress test and the constant contact of these areas with the training table, which influences changes in body surface temperature, not due to the work of the muscles but rather as a result of the friction between the body and the bench. The analyzed areas are presented in Figure 1.

The surface temperatures on both the anterior and posterior sides were assessed each time standing, in the position of abduction of the upper limbs in the brachial joints in the frontal plane. The camera was turned on 10 min before the first measurement and set at a distance of 1.5 m from the participant, perpendicular to the surface of the analyzed area. The camera was calibrated each time. During imaging, the ambient temperature and humidity were constant at the measurement site, 26 °C and 55–60%, respectively. Air temperature and relative humidity were monitored on an ongoing basis by a thermohygrometer (digital thermohygrometer, TFA Dostmann, Wertheim-Reicholzheim, Germany) and taken into account when configuring the thermal imaging camera. According to the standards of thermal imaging examinations, before taking the first image, the subjects rested in a standing position for 15 min with their upper limbs and backs exposed (elimination of the influence of clothing and thermal acclimation). Then, the swimmers were subjected to the exercise test.

The measurements were performed with an infrared digital camera FLIR E-60 (Flir Systems Inc., Wilsonville, OR, USA) with noise-equivalent temperature difference (NETD) < 0.05 °C, focal plane sensor array size of 320 × 240, and measurement uncertainty of ±2% of the overall operational temperature range. The emissivity was set for skin at 0.98 [37]. Each of the thermograms taken was subjected to detailed analysis using FLIR TOOLS software, obtaining average, minimum, and maximum temperatures from the analyzed areas.

### 2.3. Statistical Analysis

Statistical calculations were made with the use of the Statistica 13.3 software (Statistica 13 PL, StatSoft). The distribution of results was tested using the Shapiro–Wilk test. As the features of the normal distribution were confirmed, the characteristics of the analyzed variables were presented as arithmetic means and standard deviation. Student’s *t*-test was used to evaluate the significance of differences between the right and left sides of the body and the anterior and posterior sides of the body of the assessed areas. The analysis of variance and the Tukey’s HSD and Newman–Keuls post hoc tests were used to assess the changes in surface temperatures over time. The *p*-value < 0.05 was considered statistically significant.

## 3. Results

Based on the analysis of thermograms taken before, immediately after, and at the 3rd, 6th, 9th, and 12th minutes after exercise on a swimming ergometer, mean values of surface temperatures were determined from the anterior and posterior arm and forearm area, as well as from the upper and lower back area. Before physical exercise, under resting and thermal comfort conditions, the highest mean surface temperature was recorded in the posterior arm (35.4 ± 0.77 °C and 35.3 ± 0.70 °C for the right and left arm, respectively), while the lowest was recorded in the upper back (33.7 ± 1.08 °C). There was no asymmetry between the mean temperature values of the corresponding areas of the right and left upper limbs, and these differences did not exceed 0.5 °C. On the other hand, the differences in mean temperature values between the anterior and posterior arm and between the upper and lower back were found to be statistically significant (Figures 3 and 7). The mean temperature values for the assessed areas of the upper limbs and the back at individual measurement points, as well as the values of the differences between the right and left sides (∆R/L) of the corresponding areas are summarized in Table 1 and Table 2.

As a result of physical exercise, the temperature of the analyzed areas changed in all swimmers, and the size of these changes was estimated based on the calculation of the difference between the temperature values for subsequent measurement times (ΔT_before–after_; ΔT_after–3min_; ΔT_3–6min_; ΔT_6–9min_; ΔT_9–12min_). The values of these differences are summarized in Table 3. Positive values indicate a temperature decrease, while negative ones indicate its increase in relation to the value from the previous measurement time.

**Figure 2:**
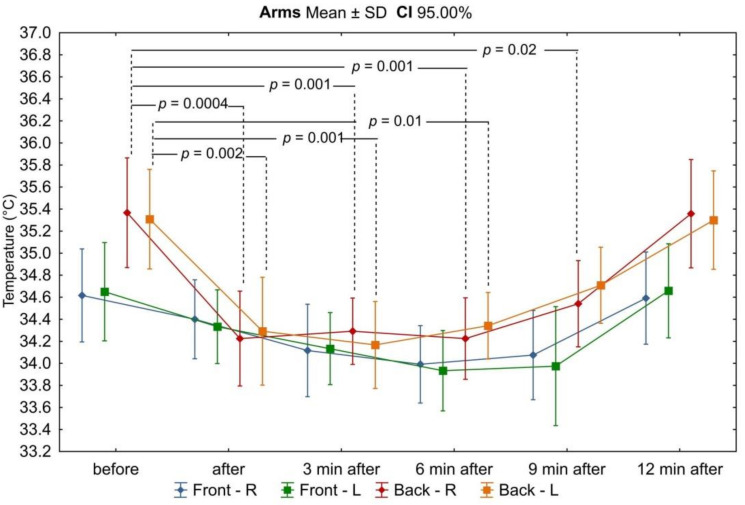
Dynamics of changes in the mean values of the surface temperature of the arms during successive measurement points before and after the exercise test. Legend: test probability level determination for Tukey’s RIR post hoc tests. Data are expressed as mean with standard deviation.

**Figure 3:**
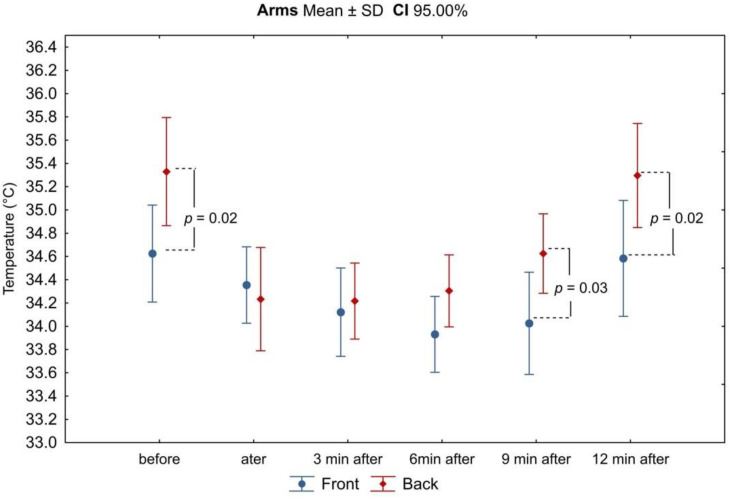
Differences in mean temperature values between the anterior and posterior surfaces of the arms during successive measurement points before and after the exercise test. Legend: the test probability level of the student *t*-test between the temperature of the anterior and posterior surfaces. Data are expressed as mean with standard deviation.

**Figure 4:**
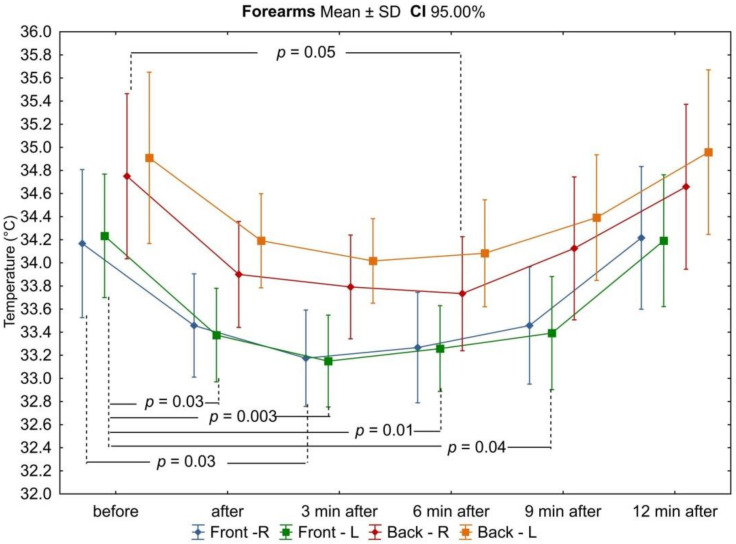
Dynamics of changes in the mean values of the surface temperature of the forearms during subsequent measurement points before and after the exercise test. Legend: test probability level determination for Tukey’s RIR post hoc tests. Data are expressed as mean with standard deviation.

**Figure 5:**
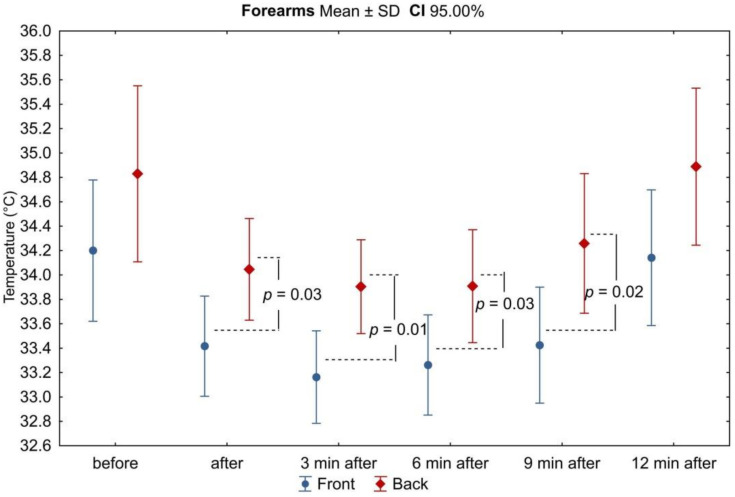
Differences in mean temperature values between the anterior and posterior surfaces of the forearms during successive measurement points before and after the exercise test. Legend: the test probability level of the student *t*-test between the temperature of the anterior and posterior surfaces. Data are expressed as mean with standard deviation.

**Figure 6:**
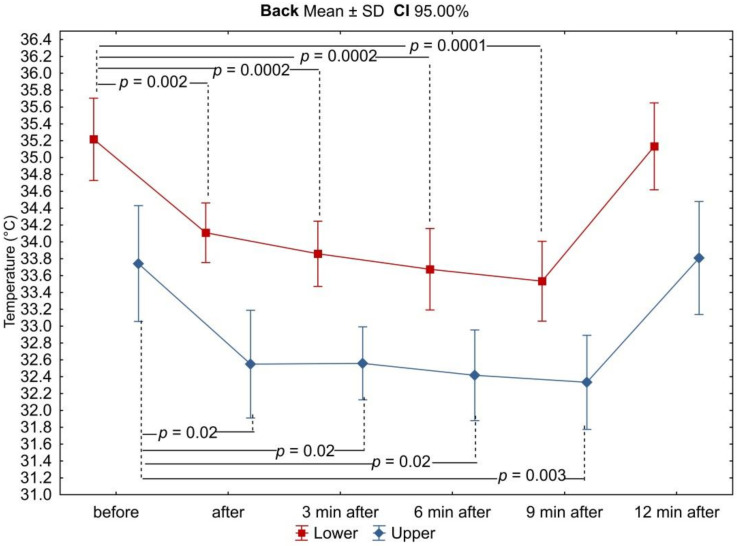
Differences in mean temperature values between the anterior and posterior surfaces of the forearms during successive measurement points before and after the exercise test. Legend: test probability level determination for Tukey’s RIR post hoc tests. Data are expressed as mean with standard deviation.

**Figure 7:**
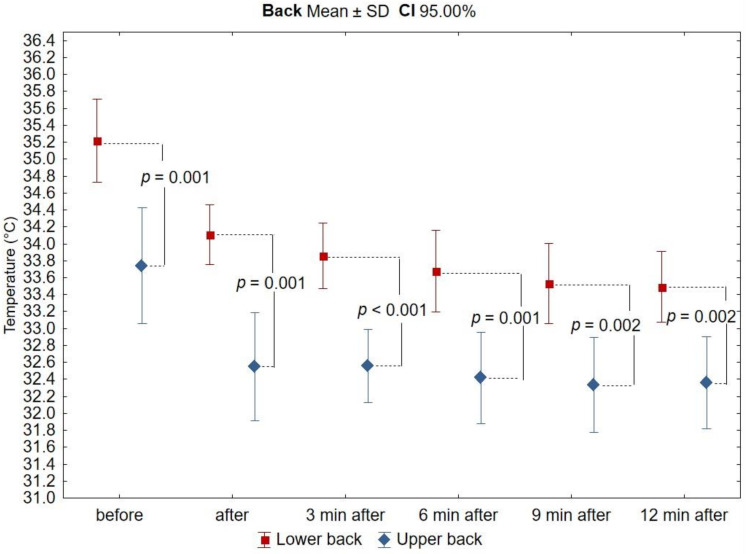
Differences in mean temperature values between the upper and lower back during successive measurement points before and after the exercise test. Legend: the test probability level of the student *t*-test between the temperature of the anterior and posterior surfaces. Data are expressed as mean with standard deviation.

In all analyzed areas, there was a decrease in surface temperature immediately after exercise, which was maintained for the right and left sides up to 3 min of observation (Table 3, Figure 2, Figure 3, Figure 4, Figure 5, Figure 6 and Figure 7). The largest drop in temperature was recorded in the posterior arms, on average by 1.2 ± 1.02 °C and by 1.0 ± 1.01 °C for the right and left arms, respectively. A similar decrease in temperature was recorded for the back region (1.2 ± 0.67 °C upper back and 1.1 ± 0.69 °C lower back). The lowest drop in temperature was observed in the anterior region of the arms (0.2 ± 0.78 °C and 0.3 ± 0.74 °C for the right and left arms, respectively) (Table 2). Immediately after exercise, the difference between the mean values of the anterior and posterior surface of the arms (Figure 3) equalized, in contrast to the area of the forearms where the difference between the mean temperatures of the anterior and posterior areas increased significantly (Figure 5). In the area of the back, both immediately after exercise and throughout the observation period, the mean value of the temperature of the upper back was significantly lower than that in the lower back (Figure 7), as under resting conditions.

In the third minute of observation after exercise, the temperature values of some analyzed areas began to increase, on average by 0.1 ± 0.43 °C and 0.1 ± 0.51 °C for the anterior regions of the right and left forearms, respectively, 0.2 ± 0.28 °C for the posterior region of the left arm, and 0.1 ± 0.45 °C for the posterior region of the left forearm. Moreover, the level of significance of differences between the mean values of the temperature of the anterior and posterior forearms (Figure 5) and the upper and lower back (Figure 7) increased. Mean values of temperature in the area of the back continued to decrease throughout the nine-minute postexercise observation period.

From 6 min after the end of exercise on the swimming ergometer, the mean temperature values of all assessed areas related to the upper limbs increased, but none of them returned to the values before exercise by the end of the observation period (Table 2, Figure 2, Figure 3, Figure 4, Figure 5, Figure 6 and Figure 7). The differences in mean temperature values between the anterior and posterior forearms remained significant (Figure 5). This situation did not change until the end of the observation period.

In the ninth minutes after exercise, a greater increase in temperature was observed in the posterior areas of the upper limbs (right arm: −0.3 ± 0.40 °C; left arm: −0.3 ± 0.37 °C; right forearm: −0.4 ± 0.44 °C; left forearm: −0.3 ± 0.40 °C) in relation to the anterior areas (−0.1 ± 0.53 °C; −0.1 ± 0.53 °C; −0.2 ± 0.29 °C; −0.1 ± 0.54 °C, respectively). The difference in mean temperature values between the anterior and posterior areas of the arms also increased, again gaining statistical significance (Figure 3).

At the last measurement point, i.e., 12 min after exercise, the temperature of all analyzed areas of the upper limbs returned to the resting values of thermal comfort. The ridge temperature has not changed.

Throughout the observation period after exercise, no statistically significant differences in mean temperature values between the right and left sides of the body were found in any of the analyzed areas of the upper limbs, and these differences did not exceed the value of 0.5 °C.

## 4. Discussion

The conducted research was aimed at assessing whether the use of thermal imaging could be effective in assessing the involvement of muscles in the exercise test on a swimming ergometer by analyzing changes in the surface temperature of the skin after exercise. It was assessed whether it would be possible to visualize and verify the symmetry of the opposite body regions involved in the effort. The findings showed a clear decrease in the surface temperature of the areas above the muscle groups involved in physical effort (i.e., the anterior and posterior areas of the arms and forearms, as well as the back region), which proves their intense work and the involvement of thermoregulatory mechanisms. The analysis of the thermograms made showed that the larger drop in body temperature recorded immediately after exercise concerned the areas that were more involved during the test (the posterior arms and the back region). A physiologically important element of physical training, especially in competitive sports, is the increase in the ability to remove heat from the body (acceleration of the sweating reaction and dynamics and minimization of the increase in internal temperature), which allows you to continue the exercise [38]. In a trained body, as a result of adaptive changes to exercise, lower increases in internal temperature and increased sweat secretion are recorded, which in turn leads to a decrease in body surface temperature [20,31,39]. Thus, the decrease in temperature in all analyzed areas of the body, recorded immediately after intense exercise, was related to the efficient activation of thermoregulatory processes by swimmers. Changes in the temperature of the body surface may indicate the load on the musculoskeletal system, provide information about the efficiency of endogenous heat dissipation systems during exercise and metabolic changes related to a return to homeostasis after exercise, and thus prove the usefulness of thermal imaging as a method of monitoring these phenomena [38]. Importantly, throughout the test period, no thermal asymmetry was recorded (in literature, differences in temperature of up to 0.5 °C between the of body surfaces are regarded as insignificant [30]) between the corresponding areas of the right and left sides of the swimmers’ bodies, which indicates that the muscles are evenly loaded during a two-minute exercise on a swimming ergometer. This result applies to the mean values of the entire study group; therefore, it may provide information on the effectiveness of training using the VASA ergometer in bilateral strengthening of the muscle groups involved in swimming, but also indicates the possibility of controlling the symmetry of the technique of swimming movements with the use of thermal imaging. This possibility is an extremely important aspect of achieving sports results, especially in sports related to swimming, where symmetrical movement technique and even generation of driving forces are the basis for success [40]. In order to confirm the result, a study should be conducted by increasing the size of the group of swimmers. When analyzing the results of previous studies, it seems that during symmetrical activities such as walking [41] or in cyclical sports (such as cycling, running, or rowing), thermal asymmetries do not occur under the conditions of proportional and economic distribution of the forces of working muscles [30,31,33]. On the assumption that swimming is also a cyclical sport, no thermal asymmetries should be recorded among technically trained swimmers after a physical exercise related to this activity.

It is known that under physiological conditions, in the absence of disturbances in the musculoskeletal system and changes related to the occurrence of inflammation, the human body is thermally symmetrical, and differences above 0.5 °C between opposite sides may indicate various types of dysfunction [42,43,44]. In this study, there was no asymmetry of both mean and individual temperature values between the analyzed areas of the right and left side of the subjects’ bodies at rest, under thermal comfort conditions. However, a detailed analysis of the individual changes in the body temperature of the subjects showed transient and temporary differences in the temperature values of the corresponding areas of the upper limbs, amounting to >0.5 °C (thus considered physiologically significant), which appeared at different times of observation after physical exercise. This may be evidence of the high sensitivity of the thermal imaging method in the individual analysis of the involvement of muscular effort, or in the assessment of the efficiency of thermoregulatory processes in individual subjects. Despite taking into account all the recommended procedures for measuring the surface temperature of the body using the thermal imaging method, the temporariness and transient nature of the recorded individual asymmetries confirm the individually specific thermal response to physical exercise. Therefore, the sensitivity of the thermal imaging method requires extremely scrupulous observance of the measurement procedures in order to eliminate the influence of external factors on the result of the body surface temperature measurement, and the interpretation of the results should be based on the reference to contralateral values or, as was the case in the presented study, also changes over time within the same area. There are no reports in the available literature concerning the analysis of changes in the body surface temperature of the areas involved in dry-land training over time, corresponding to the technical swimming movements. The studies conducted so far focused mainly on the analysis of the mean values of changes in the surface temperature of the body after the swimmers’ exercise in the water environment [13,45], without describing individual changes, but presenting only the mean values of the resting temperature and its changes 15 min after swimming a specific distance front crawl and breaststroke. The study by Zaidi’s research team, on the other hand, showed the results for the change in temperature of different areas of the body of one swimmer, depending on the style of swimming he used to cover the distance of 100 m in the pool [46]. These authors did not analyze the symmetry of surface temperature changes between the right and left sides of the body, but only changes over time, in the temperature of individual areas and the mean temperature from all areas of the body immediately after swimming 100 m in each of the four analyzed swimming styles (butterfly, backstroke, breaststroke, and freestyle). The results of the above study showed that all swimming styles caused a significant increase in skin temperature, and its value depended on the specific style and the assessed body area. The temperature of the lower limbs increased the most after swimming the designated distance in the breaststroke style. On the other hand, the highest increase in surface temperature in the regions of the back and upper limbs, and the highest mean temperature for all areas, was recorded for the backstroke style. It should be noted that the reported increase in the mean temperature of the whole body area (by 2.16 °C for the butterfly, 2.56 °C for the backstroke, 1.78 °C for the breaststroke, and 2.00 °C for the freestyle, respectively) was related to the baseline body surface temperature lowered by 10 min acclimation in water at a temperature of 27 °C [46]. The values of the surface temperature of the body measured by the thermal imaging method among people performing physical exercise in the water and land environments cannot be compared with each other, because the environment itself significantly affects the obtained results. In water, the main heat exchange mechanisms are conduction and convection, so the heat loss in water is about 20 times higher than the heat exchange between the skin and air [47]. However, despite the training environment being different to the one used in our own research, Novotny et al. did not observe any thermal asymmetry within the analyzed areas of the right and left sides of the body, both under resting conditions and after 15 min exercise in water [13,45].

The ability to analyze the individual body surface temperature of athletes in real time using thermal imaging techniques allows you to control the body’s response to physical workload. The data obtained in this way can be used to assess the degree of involvement of selected muscle groups during exercise as a tool for coaches to assess not only the dynamics of the underlying surface temperature during exercises, but also to assess the symmetry of muscle activity. Further, the data provide valuable information in terms of assessing the effectiveness of training and give the opportunity to determine the individual performance and capabilities ofan athlete [48]. Thermal imaging is also a potential tool to identify early local skin vasomotor changes in an objective evaluation of an athlete’s heat production to prevent injuries and estimate workload [26]. These aspects seem to be important especially among swimmers who, on the one hand, should make the most symmetrical effort possible both in terms of the technique of movement and the value of the generated muscular strength, and on the other hand, during intense training, are exposed to the occurrence of injuries resulting mainly from overload of the musculoskeletal system.

We are well aware that a relatively small study group consisting only of men is a limitation of the research. It would be worth verifying the obtained results by conducting research among a larger group of swimmers, taking into account the muscular activity corresponding to possibly various swimming styles and the participation of women in the study. 

## 5. Conclusions

The thermal imaging method is a sensitive tool in the individual analysis of the involvement of muscles and in assessing the efficiency of thermoregulatory processes of individual swimmers during dry-land training on a swimming ergometer. The VASA swimming ergometer seems to be an effective tool to improve the swimming technique in terms of its symmetry (freestyle).

## Figures and Tables

**Table 1:** Characteristics of mean values (±SD) of surface temperatures for the analyzed body regions (°C).

	Body Area		Mean ± SD [°C]		∆R/L	Student’s *t*-test *p* R/L Side	
			Right Side	Left Side		t	*p*
T_befor_	A	front	34.6 ± 0.66	34.7 ± 0.67	0.2 ± 0.15	−0.2454	0.8084
		back	35.4 ± 0.77	35.3 ± 0.70	0.2 ± 0.13	0.1932	0.8485
	FR	front	34.2 ± 1.01	34.2 ± 0.84	0.3 ± 0.20	−0.1759	0.8620
		back	34.8 ± 1.13	34.9 ± 1.17	0.3 ± 0.21	−0.3383	0.7384
T_after_	A	front	34.4 ± 0.54	34.3 ± 0.53	0.2 ± 0.19	0.1909	0.8504
		back	34.2 ± 0.67	34.3 ± 0.76	0.3 ± 0.20	−0.3404	0.7368
	FR	front	33.5 ± 0.70	33.4 ± 0.64	0.3 ± 0.20	0.3041	0.7639
		back	33.9 ± 0.72	34.2 ± 0.64	0.4 ± 0.28	−1.0467	0.3066
T_3min_	A	front	34.1 ± 0.69	34.1 ± 0.55	0.3 ± 0.21	0.0658	0.9482
		back	34.3 ± 0.44	34.2 ± 0.62	0.2 ± 0.25	0.5294	0.6019
	FR	front	33.2 ± 0.66	33.2 ± 0.63	0.4 ± 0.27	0.0955	0.9248
		back	33.8 ± 0.71	34.0 ± 0.57	0.3 ± 0.35	−0.8557	0.4014
T_6min_	A	front	34.0 ± 0.55	33.9 ± 0.57	0.3 ± 0.17	0.2534	0.8023
		back	34.2 ± 0.58	34.4 ± 0.47	0.3 ± 0.28	−0.7306	0.4727
	FR	front	33.3 ± 0.75	33.3 ± 0.58	0.3 ± 0.16	0.0303	0.9761
		back	33.7 ± 0.78	34.1 ± 0.73	0.4 ± 0.32	−1.1390	0.2669
T_9min_	A	front	34.1 ± 0.64	34.0 ± 0.85	0.3 ± 0.48	0.3265	0.7472
		back	34.5 ± 0.62	34.7 ± 0.54	0.4 ± 0.30	−0.7030	0.4894
	FR	front	33.5 ± 0.80	33.4 ± 0.77	0.4 ± 0.26	0.2082	0.8370
		back	34.1 ± 0.97	34.4 ± 0.86	0.3 ± 0.26	−0.7129	0.4834
T_12min_	A	front	34.6 ± 0.66	34.7 ± 0.67	0.2 ± 0.15	−0.2454	0.8084
		back	35.4 ± 0.77	35.3 ± 0.70	0.2 ± 0.13	0.1932	0.8485
	FR	front	34.2 ± 0.97	34.2 ± 0.90	0.3 ± 0.20	0.0655	0.9484
		back	34.7 ± 1.12	35.0 ± 1.12	0.3 ± 0.21	−1.6768	0.1077

Abbreviations: A—arms; FR—forearms; B—back.

**Table 2:** Characteristics of the median values (±SD) of the distribution of surface temperatures in the back area (°C).

Body Area	Me ± SD [°C]					
	T_berofe_	T_after_	T_3min_	T_6min_	T_9min_	T_12min_
UB	33.7 ± 1.08	32.6 ± 1.01	32.6 ± 0.68	32.4 ± 0.85	32.3 ± 0.88	32.3 ± 0.81
LB	35.2 ± 0.77	34.1 ± 0.56	33.9 ± 0.61	33.7 ± 0.76	33.5 ± 0.75	33.5 ± 0.96

Abbreviations: UB–upper back; LB–lower back.

**Table 3:** Characteristics of changes in mean values (±SD) of surface temperature of the assessed areas over time (°C).

Body Area			Mean ± SD [°C]					ANOVA		Power
			ΔT_before–after_	ΔT_after—3min_	ΔT_3–6min_	ΔT_6–9min_	ΔT_9–12min_	F	*p*	for α = 0.05
Arms	front	R	0.2 ± 0.78	0.3 ± 0.43	0.1 ± 0.50	−0.1 ± 0.53	−0.5 ± 0.56	2.304	0.054	0.806
		L	0.3 ± 0.74	0.2 ± 0.33	0.2 ± 0.36	−0.1 ± 0.53	−0.7 ± 0.87	2.941	0.019	0.958
	back	R	1.2 ± 1.02	0.1 ± 0.43	0.1 ± 0.27	−0.3 ± 0.40	−0.8 ± 0.71	8.349	<0.0001	1.000
		L	1.0 ± 1.01	0.1 ± 0.41	−0.2 ± 0.28	−0.3 ± 0.37	−0.6 ± 0.54	7.536	<0.0001	0.999
Forearms	front	R	0.7 ± 1.20	0.3 ± 0.39	−0.1 ± 0.43	−0.2 ± 0.29	−0.8 ± 0.82	3.628	0.006	0.989
		L	0.9 ± 1.06	0.2 ± 0.41	−0.1 ± 0.51	−0.1 ± 0.54	−0.8 ± 0.66	5.166	<0.0001	0.998
	back	R	0.9 ± 0.77	0.1 ± 0.43	0.1 ± 0.33	−0.4 ± 0.44	−0.5 ± 0.85	2.776	0.025	0.927
		L	0.7 ± 0.93	0.2 ± 0.48	−0.1 ± 0.45	−0.3 ± 0.40	−0.6 ± 0.73	2.670	0.029	0.976
Back	upper		1.2 ± 0.67	0.0 ± 0.63	0.1 ± 0.47	0.1 ± 0.25	0.03 ± 0.4	4.286	<0.001	0.998
	lower		1.1 ± 0.69	0.3 ± 0.40	0.2 ± 0.33	0.1 ± 0.46	0.04 ± 0.3	10.615	<0.0001	1.000

## Data Availability

Not applicable.
